# Supplementary material for: Micronutrient intake and prevalence of micronutrient inadequacy among women (15-49 y) and children (6-59 mo) in South Kivu and Kongo Central, Democratic Republic of the Congo (DRC)
Source: PLoS One. 2020 Jun 12;15(6):e0223393. doi: 10.1371/journal.pone.0223393 (PMC7292409; doi:10.1371/journal.pone.0223393)
Supplement: Attachment — Submitted filename: Plos One_Response to Reviewers.docx [file pone.0223393.s001.docx]

**PlosOne_Reviewer Comments**

| **Additional Journal Requirements** | **Response** |
| --- | --- |
| 1. When submitting your revision, we need you to address these additional requirements. Please ensure that your manuscript meets PLOS ONE's style requirements, including those for file naming. The PLOS ONE style templates can be found at   <http://www.journals.plos.org/plosone/s/file?id=wjVg/PLOSOne_formatting_sample_main_body.pdf>  and  <http://www.journals.plos.org/plosone/s/file?id=ba62/PLOSOne_formatting_sample_title_authors_affiliations.pdf>   2. Please include additional information regarding the survey or questionnaire used in the study and ensure that you have provided sufficient details that others could replicate the analyses. For instance, if you developed a questionnaire as part of this study and it is not under a copyright more restrictive than CC-BY, please include a copy, in both the original language and English, as Supporting Information. Moreover, please include more details on how the questionnaire was pre-tested, and whether it was validated. 3. Please consider including more details on the qualitative methods used during the focus group meetings (for  qualitative research, we suggest consulting the COREQ guidelines: <http://intqhc.oxfordjournals.org/content/19/6/349>. 4. We note that you have stated that you will provide repository information for your data at acceptance. Should your manuscript be accepted for publication, we will hold it until you provide the relevant accession numbers or DOIs necessary to access your data. If you wish to make changes to your Data Availability statement, please describe these changes in your cover letter and we will update your Data Availability statement to reflect the information you provide. | 1. We thank the journal for this comment and have done so. 2. We will include a copy of the questionnaire in the repository in both English and French. 3. We thank the journal for this comment and have included details around the qualitative aspect of the survey in line 165-176. 4. We still intend to make our data publicly available upon acceptance for publication. |
| **Reviewer #1 Comments** |  |
| The objective of this study was to determine the micronutrient intakes and prevalence of inadequate intakes in two provinces of the Democratic Republic of the Congo. My main recommendation is that statistical comparisons should be made so that statements can be made about differences in intake. It seems that differences between provinces and age groups would be appropriate.  Secondly, the authors should comment on the discrepancy of iron deficiency estimates based on the biochemical data. In the abstract and throughout the manuscript, the authors indicate that there is a low prevalence of iron deficiency in women of reproductive age and their children (~5% and ~3% in women of reproductive age and children, respectively). However, this statement is based on ferritin concentrations that are reported in the primary study (PMID: 26901219). sTfR was also measured in the primary study and using sTfR, the prevalence of iron deficiency in these 2 populations was ~20% and ~63%, respectively, which would be more in line with the dietary data.  **Additional suggestions are included below:**   1. The following phrases in the abstract may be unclear to the reader without additional context and should be removed: “(with repeats)”, “(10% bioavailability)”, and “However, any change to the supply of red palm oil would dramatically reduce population vitamin A intakes..” 2. **Line 45-46:** include population. WRA? 3. **Line 48**: A percentage is given for WRA, but not children. 4. Is it safe to say that iron biofortified beans may be warranted based on dietary intake data alone? This statement conflicts with the low rates of iron deficiency based on ferritin concentration. 5. Citations are missing and should be included (e.g., line 65-66, 79-80). 6. **Line 71-78**: Is this data from the parent study? If so, it should be cited. 7. Be consistent with standard deviations and standard errors in the tables. 8. Anytime comparisons are made, statistics should be performed (e.g., line 247-248). 9. Statistics should be performed in order to compare intakes across provinces, and possible age groups. 10. If prevalence of micronutrients besides iron, zinc, and vitamin A are shown in Tables 2-5, should food sources of these nutrients also be shown? 11. Is it accurate to say that the results from the dietary assessment survey “mimic” the findings from the biochemical survey? 12. Per PLOS ONE policy, a comment needs to be made regarding data availability. | We have made the suggested statistical comparisons between provinces. We lacked power to make comparisons within age bands especially when aggregated by province  We appreciate this comment. We have included the rationale for using the ferritin concentrations instead of sTfR in line 438-448. Subsequent analysis of our biochemical findings has revealed a high prevalence of inherited blood disorders in our sample. sTfR concentrations were significantly associated with glucose-6-phosphate-dehydrogenase variant genotypes, whereas haemoglobin and ferritin were not. Thus, ferritin adjusted for inflammation is the most suitable biomarker of iron status in this population.   1. We have removed confusing language and provided more context around the phrase “However, any change to the supply of red palm oil would dramatically reduce population vitamin A intakes” in line 55-57. 2. Thank you for catching this oversight. We have specified the population in line 45-46. 3. We have provided a percentage the children in lines 48-51. 4. We agree and have removed the sentence recommending iron biofortified beans. 5. Citations have been provided in line 67-69, 81-82. Apologies for the oversight. 6. Yes, the parent study has been cited in line 73-80. 7. Standard deviations have been removed from Table one to be in line with the rest of the results, only SE have been used 8. We have provided statistical comparisons as requested. 9. Statistical comparisons have been made for differences in intake and prevalence of inadequacy between provinces. This has been described in the methods in lines 241-244 and shown in the results in Tables 2-5. 10. We didn’t include food sources of all nutrients if they couldn’t be compared to biochemical analysis. Line 343-346. We only measured B12, folate, iron, zinc, and vitamin A status. Since there was no evidence of B12 deficiency or folate deficiency from dietary and biochemical measures we did not investigate further. 11. No, it is not. We have revised this statement to say “results of our dietary assessment are consistent with the biochemical findings, except for iron”. Line 500-501 12. We have included a data sharing statement along with DOI link. |
| **Reviewer #2 Comments** |  |
| The study investigated the prevalence of inadequate intakes of selected nutrients in two provinces of DRC to explain findings from biochemical assessment and justify need for proposed dietary interventions. The manuscript is well written, and the following comments are suggestions to improve the content.   1. **Abstract**: methods have not been adequately described in the abstract. Authors should provide a brief description of how the prevalence of inadequate intakes was estimated. 2. **Line 166:** How does use of the USDA nutrient database affect the nutrient composition of the foods. Did they use raw foods, or did they also include minimally processed food components also. Some items in the USDA nutrient database may be fortified with vitamins and minerals. Authors should indicate approaches they used to ensure the best choices of the foods from the USDA database were selected to minimize over or underestimation of nutrient contents. 3. **Line 250:** 32 % is the average for all subjects. The current presentation suggests that each of the two districts had a prevalence of inadequacy of 32 %. I suggest the authors delete “32 %”. 4. **Table 6:** Antelope does not seem to be providing any of the nutrients, yet it is listed among the top sources of the three micronutrients. Or maybe the value is missing. 5. **Line 388-390**. I wonder if the use of the EFSA physiological requirements for zinc played a role in the very high prevalence of inadequate zinc intakes in the populations studied. How would the results compare if the authors had used the US Institute of Medicine (IOM) physiological requirement values instead, since for all the other analysis they used EAR values from US IOM? Could they have used the IOM physiological requirements and applied appropriate bioavailability estimates and compare results? 6. **Line 396**: Animal source foods are rich sources of Vitamin B12, zinc and iron. While vitamin B12 is primarily from animal source foods and fortified foods, plant foods also provide zinc and iron. It is therefore unexpected to find a huge prevalence of inadequate zinc and iron intakes and high level of deficiencies of these two nutrients while vitamin B12 intake is highly sufficient. The authors need to address this discrepancy. | 1. We have included an explanation on how prevalence of inadequate intakes was estimated in line 44-45 2. We used raw foods and applied USDA retention factors to account for water and nutrient losses due to cooking. Line 181-182. The USDA database made up less than 10% of the in our composition database and these were food infrequently consumed and not fortified 3. We have made the deletion.      4. We thank the reviewer for the comment and have corrected the error in Table 6. 5. EFSA requirements are the most recent and similar to the IOM requirements, thus we expect little difference in prevalence of inadequacies when using IOM vs EFSA. Rather, the other requirements that are frequently used are IZINCG requirements, and these are very low compared to IOM and EFSA resulting in an underestimate of the prevalence of zinc inadequacy. We convened a consultation of experts in 2012 to resolve these inconsistencies, and it was determined that there are methodological flaws in both, the more serious affecting IZINCG. The corrected values for women are closer to IOM’s, and even closer to EFSA’s.   References:  Moursi M. 2014. “Human Zinc Requirements: Report of the HarvestPlus Consultation on Physiological and Dietary Zinc Requirements.” July 24, 2012 HarvestPlus. NIH Conference Room 5A01 6100 Executive Boulevard Rockville, Maryland 20892, USA.  Accessed online: <https://www.harvestplus.org/sites/default/files/Report%20Zinc%20consultation_12%2010%2012.pdf>  Hambidge KM, Miller LV, Krebs NF. Physiological requirements for zinc. Int J Vitam Nutr Res. 2011 Jan;81(1):72-8.  Gibson RS, King JC, Lowe N. A Review of Dietary Zinc Recommendations. Food Nutr Bull. 2016 Dec;37(4):443-460. Epub 2016 Jun 16. Review.   6. We have addressed this discrepancy in line 427-430. |
