# Supplementary material for: Micronutrient intake and prevalence of micronutrient inadequacy among women (15-49 y) and children (6-59 mo) in South Kivu and Kongo Central, Democratic Republic of the Congo (DRC)
Source: PLoS One. 2020 Jun 12;15(6):e0223393. doi: 10.1371/journal.pone.0223393 (PMC7292409; doi:10.1371/journal.pone.0223393)
Supplement: Attachment — Submitted filename: Response to Reviewers.docx [file pone.0223393.s002.docx]

**PlosOne_Reviewer Comments**

| **Reviewer #2 Comments** |  |
| --- | --- |
| 6. The authors have adequately responded to all previous comments. I only have a minor comment on the reported values in some of the tables as follows: For tables 2-5, under the prevalence of inadequate intakes, the authors have not reported the SE for some of the values. This should be corrected or the reasons for not being able to estimate these values should be mentioned as part of the table footnotes. | 6. We thank the reviewer for this comment. The probability approach was used to estimate prevalence of inadequacy for iron rather than the cut-point method. IMAPP does not provide a SE for probability of inadequacy when using this approach. For Vitamin B_12_, PCSIDE software was used to estimate prevalence of inadequacy as there were too few replicates and it appeared episodically in the diet. PC-SIDE does not report SE for prevalence of inadequacy estimates. These explanations have been added in the footnotes for tables 2-5. |
