# Supplementary material for: Micronutrient intake and prevalence of micronutrient inadequacy among women (15-49 y) and children (6-59 mo) in South Kivu and Kongo Central, Democratic Republic of the Congo (DRC)
Source: PLoS One. 2020 Jun 12;15(6):e0223393. doi: 10.1371/journal.pone.0223393 (PMC7292409; doi:10.1371/journal.pone.0223393)
Supplement: Attachment — Submitted filename: rebuttal.docx [file pone.0223393.s003.docx]

1. How was having “no serious and health conditions or chronic diseases”, one of the inclusion criteria, operationalized for this study? This information might help explain the minimal evidence of iron deficiency anemia reported from biochemical assessments.

**Response:** This is a good point and likely an oversight on our part. Although this was an exclusion criterion and asked as a question for all potential participants nobody was excluded because of this. The mothers were primarily a young population and had not developed chronic disease (i.e. non-communicable disease). None of the young children had developed chronic disease. On the other hand, there was a high prevalence of Malaria especially in Kongo Central, but this was asymptomatic, and nobody was excluded

The following has been added at line 247 “No mother/ child dyad was excluded due to the presence of chronic disease.”

2. The study notes a discrepancy between dietary and biochemical assessment of iron status. They state that this discrepancy might be due to the use of nutrient reference values not suitable for a population with a high consumption of starchy roots and tubers. It will be good to expand on this plausible explanation with information on the mechanism by which they think the intake of starchy roots and tubers affect iron absorption and hence iron status.

**Response:** Again, this is our mistake we do not to mean suggest that requirements (nutrient reference values) are incorrect but rather the assumptions on the bioavailability of iron may be wrong, in our case we used 10% and 7.5% . Animal based foods are the best source of bioavailable iron and plant sources are not as good. In high income countries we might use a bioavailability percentage of 17% whereas in a country where cereal grains are the main source of iron an iron bioavailability of 5-7% may be more appropriate. The lower the bioavailability the more iron that is required to meet recommendations. For example, if the nutrient reference value is 10 and if we assume a 17% bioavailability less iron is needed in the diet than if the bioavailability is 7%. All we are saying here is we do not know the bioavailability of iron in the Congolese diet and this may be a source of the discrepancy.

We have added the following at line 462

As was highlighted by Verbowski et al, the reason for the discrepancy may be due to the use of bioavailability values for iron that are not suitable for the Congolese diet where starchy roots and tubers contribute roughly 40% of dietary energy intake. For example, for women in Kongo Central, if a 10% bioavailability for iron is assumed 29% of women have inadequate iron intakes but if 7.5% is used these increases to 52%. The bioavailability of iron ranges between <7.5% to > 17% depending on source. We do not know the overall bioavailability of iron in the Congolese diet.
